# Voltammetric Kinetic Studies of Electrode Reactions: Guidelines for Detailed Understanding of Their Fundamentals

**DOI:** 10.1021/acs.jchemed.2c00944

**Published:** 2022-12-27

**Authors:** Joaquín González, Eduardo Laborda, Ángela Molina

**Affiliations:** Departamento de Química Física, Facultad de Química, Regional Campus of International Excellence “Campus Mare Nostrum”, Universidad de Murcia, 30100Murcia, Spain

**Keywords:** electrode kinetics, Tafel analysis, Koutecký−Levich plot, ultramicroelectrodes, rotating disc electrode

## Abstract

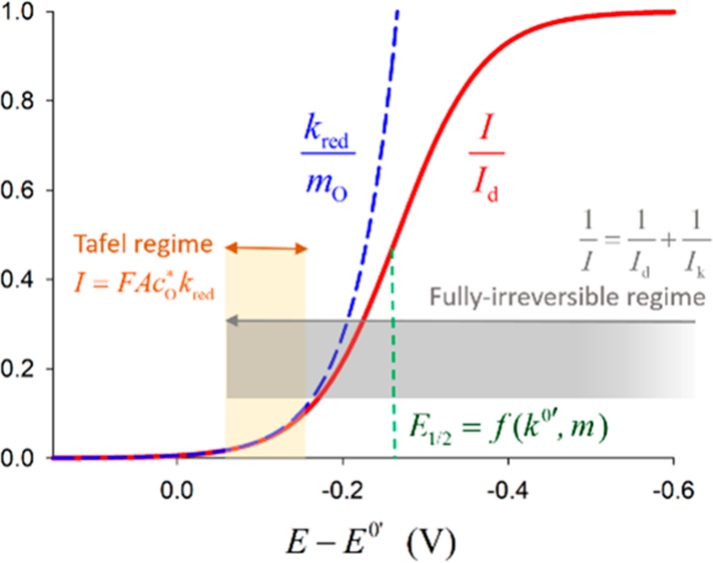

Theoretical and practical foundations of basic electrochemical concepts of heterogeneous charge transfer reactions that underline electrochemical processes are presented for their detailed study by undergraduate and postgraduate students. Several simple methods for calculating key variables, such as the half-wave potential, limiting current, and those implied in the kinetics of the process, are explained, discussed, and put in practice through simulations making use of an Excel document. The current–potential response of electron transfer processes of any kinetics (i.e., any degree of reversibility) are deduced and compared for electrodes of different size, geometry, and dynamics, namely: static macroelectrodes in chronoamperometry and normal pulse voltammetry, and static ultramicroelectrodes and rotating disc electrodes in steady state voltammetry. In all cases, a universal, normalized current–potential response is obtained in the case of reversible (fast) electrode reactions, whereas this is not possible for nonreversible processes. For this last situation, different widely used protocols for the determination of the kinetic parameters (the mass-transport corrected Tafel analysis and the Koutecký–Levich plot) are deduced, proposing learning activities that highlight the foundations and limitations of such protocols, as well as the influence of the mass transport conditions. Discussions on the implementation of this framework and on the benefits and difficulties found are also presented.

## 1 Introduction

Electrochemistry has become ubiquitous in a great number of practical applications, specifically in those involved in the generation, conversion, and storage of electrical energy.^1^ Indeed, electrochemical reactions underlie many of the technologies against climate change (e.g., batteries^2,3^), as well as water management (e.g., electrochemical remediation^4^), electrosynthesis of new materials,^5,6^ or biological processes (e.g., electron transport chains^7^), among others. However, such advances have revealed that initiatives in fundamental research in electrochemistry are unfortunately undermatched.^1,8^

In line with the above, although electrochemical thermodynamics is still well represented in the undergraduate curriculum, the teaching of the kinetic and mass transport aspects of electrochemical phenomena and techniques and their underlying laws have decreased their presence in the curriculum and textbooks of undergraduate and graduate students, as highlighted in the literature.^9−12^ The knowledge that current students have about these topics should be sound given the need for understanding the kinetics of electron transfer reactions for a thoughtful study of emerging materials that improve the performance of batteries and fuel cells, which is frequently limited by the rate of such reactions.

In a paradoxical way, the above contrasts with the fact that electrochemical techniques are relatively easy to implement so that students are able to carry out a variety of electrochemical experiments in a direct way after a short training period.^13^ This has a “dark side” since the mere application of instrumental techniques without previous understanding of the underlying phenomena is evidently undesirable. In this sense, there is the risk that Electrochemistry becomes a “test technology”, that is, a set of recipes for carrying out different protocols relative to electrochemical processes but without a solid fundamental framework that enables the adequate analysis and discussion of the results obtained beyond a “trial and error” paradigm.^14^ This situation could be amended by introducing some fundamental aspects of the kinetics of the electrochemical response in the Physical Chemistry Curriculum. Unfortunately, this is usually associated with awkward differential equations and obscure concepts that are hard to understand for undergraduate students (even for postgraduate ones!). This could be related with the fact that electrochemical processes have a heterogeneous nature since they occur at the electrode–solution interface, and therefore, their analysis requires 'more than one coordinate' mathematics. An additional problem with the teaching of electrochemistry is that the student needs to go beyond Chemistry since electrical variables, such as current and potential, also play a crucial role (an example of this issue is how the role of the electrical potential and its relationship with the Gibbs free energy are frequently problematic concepts for nonspecialists in Electrochemistry).^15,16^

In practice, three variables define the electrochemical behavior of a system: potential, current, and time. Initially, the interplay between them can be perceived as complex and difficult by students and teachers. Nevertheless, it is possible to carry out a simple yet accurate discussion of the basics of electrochemical responses, which can be very beneficial for the deep understanding of these processes.^17−20^

In this work, a simple theoretical framework and different activities for undergraduate and postgraduate students are presented, which allow for tackling very relevant electrochemical concepts and putting into practice several methods for calculating the variables implied in the kinetics of the process under study. Since the topics under study here cannot be considered as part of a general chemistry curriculum, it is necessary that the students acquire some prior knowledge related to physical chemistry in order to achieve an adequate comprehension of the topic. Thus, they should have studied chemical kinetics, the basics of solvation and mass transport phenomena, and notions about electrostatics.^10^ Depending on the specificities of each country, high level undergraduate or postgraduate students can meet such prerequisites.

**Figure 1:**
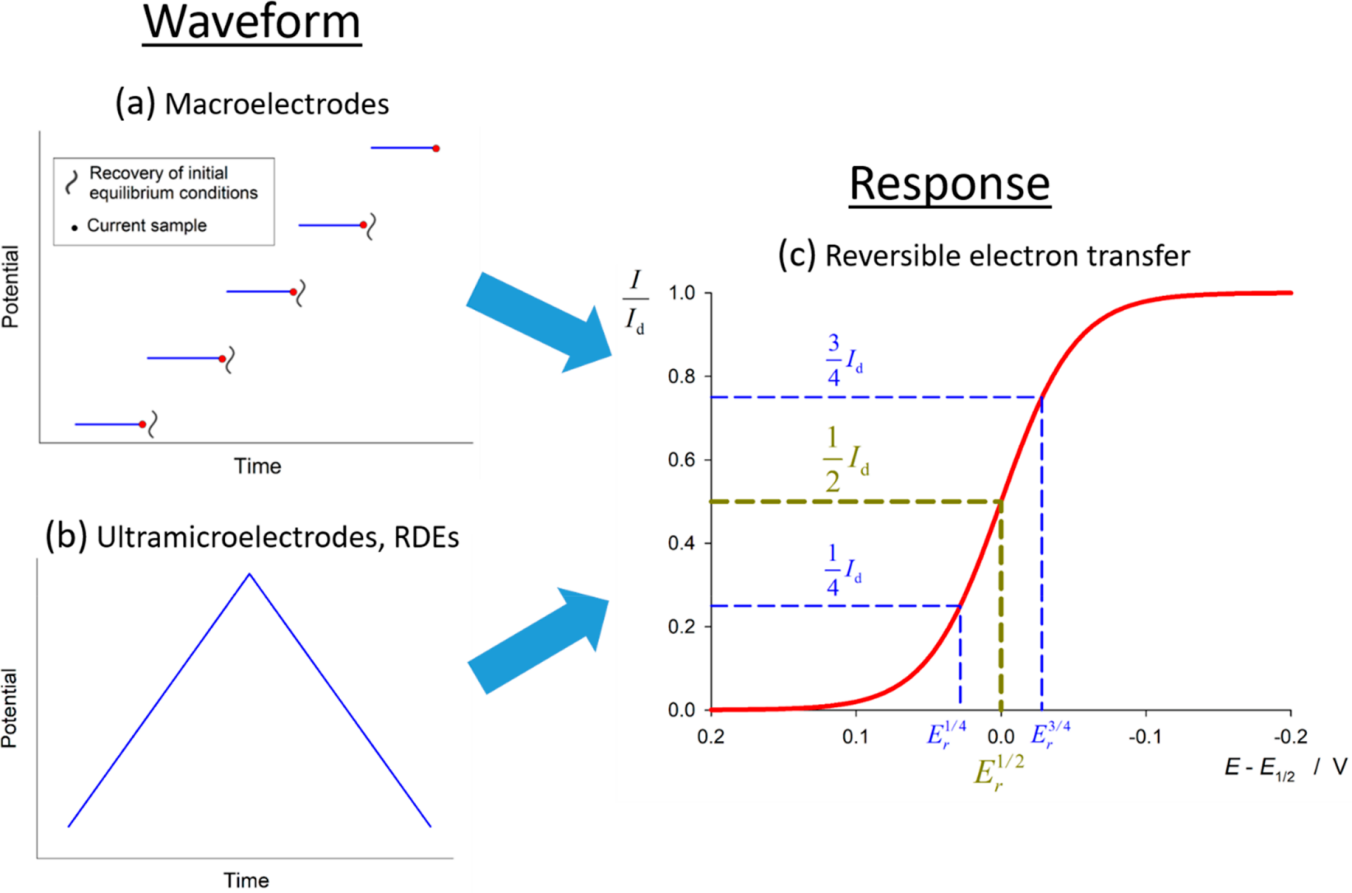
(a, b) Applied waveforms in normal pulse voltammetry (macroelectrodes) and steady state voltammetry (ultramicroelectrodes and RDEs). (c) Universal current–potential response of reversible electrode reactions. Note that waveforms a and b give rise to a single current–potential curve in the case of ultramicroelectrodes and RDEs while, in the case of macroelectrodes, waveform b gives rise to the well-known transient “duck-shaped” curve (not shown) corresponding to cyclic voltammetry.

To accomplish the objectives discussed above, simple mathematical relationships between the dependent (the current measured) and independent (the applied potential) variables of potential controlled techniques^21^ are studied and simulations are made with an Excel sheet. By means of the introduction of mass transport coefficients, exact or very accurate expressions are derived for the current–potential response of electrode reactions of any reversibility in a great variety of situations (see Figure 1): a single potential step (i.e., chronoamperometry and constant potential voltammetry) at macroelectrodes (at least of millimetric size), and any time-variable potential perturbation (typically, a triangular waveform) at electrodes of micrometric size or at rotating disc electrodes (RDEs^22^) (i.e., steady state voltammetry).^19^

With the above knowledge, it is expected that the student will be able to understand in a rational and easy way, from simple relationships between current and potential, the definition of important and most used concepts in any electrochemical study, including the well-known and more sophisticated techniques of cyclic and square wave voltammetries. Such concepts, which should be considered as the main intended learning outcomes, are the half-wave potential (dependent on the kinetics of the process and the characteristics of the diffusion conditions) and the limiting current (independent of the charge transfer kinetics).

First, the case of reversible electron transfers is considered, demonstrating that a universal current–potential response exists—regardless of the size and geometry of the electrode—provided that the adequate current normalization and potential reference potential are selected (see Figure 1). Then, the kinetic study of nonreversible electrode reactions is discussed, showing that the previous behavior cannot be found and indicating protocols for the extraction of kinetic parameters: the standard heterogeneous rate constant, *k*^0^′, and the transfer coefficient, α.

For nonreversible electrode reactions, the foundations, applicability, and limitations of widely used methods such as the mass-transport corrected Tafel analysis^23^ and the Koutecký–Levich plots for rotating disc electrodes (1/*I* vs ω^–1/2^)^19,24,25^ are compared and discussed. All these approaches are based on linearizing the current–potential response, which is an advantageous and ubiquitous practice for simple, rapid, and accurate scientific data analysis. Nevertheless, as will be discussed in this work, there are situations where, due to not-very-slow electrode kinetics and/or to complex reaction mechanism (beyond the scope of this work),^26^ the protocols mentioned above are not applicable and abusing their use can lead to significant errors in the values obtained for the kinetic parameters.

The theoretical framework, along with introductory activities related to the electrode kinetics and mass transport, will be presented, followed by discussions about the implementation and the assessment of the learning degree and the main difficulties of the students.

## 2 Theoretical Framework. Current–Potential Response

**Figure 2:**
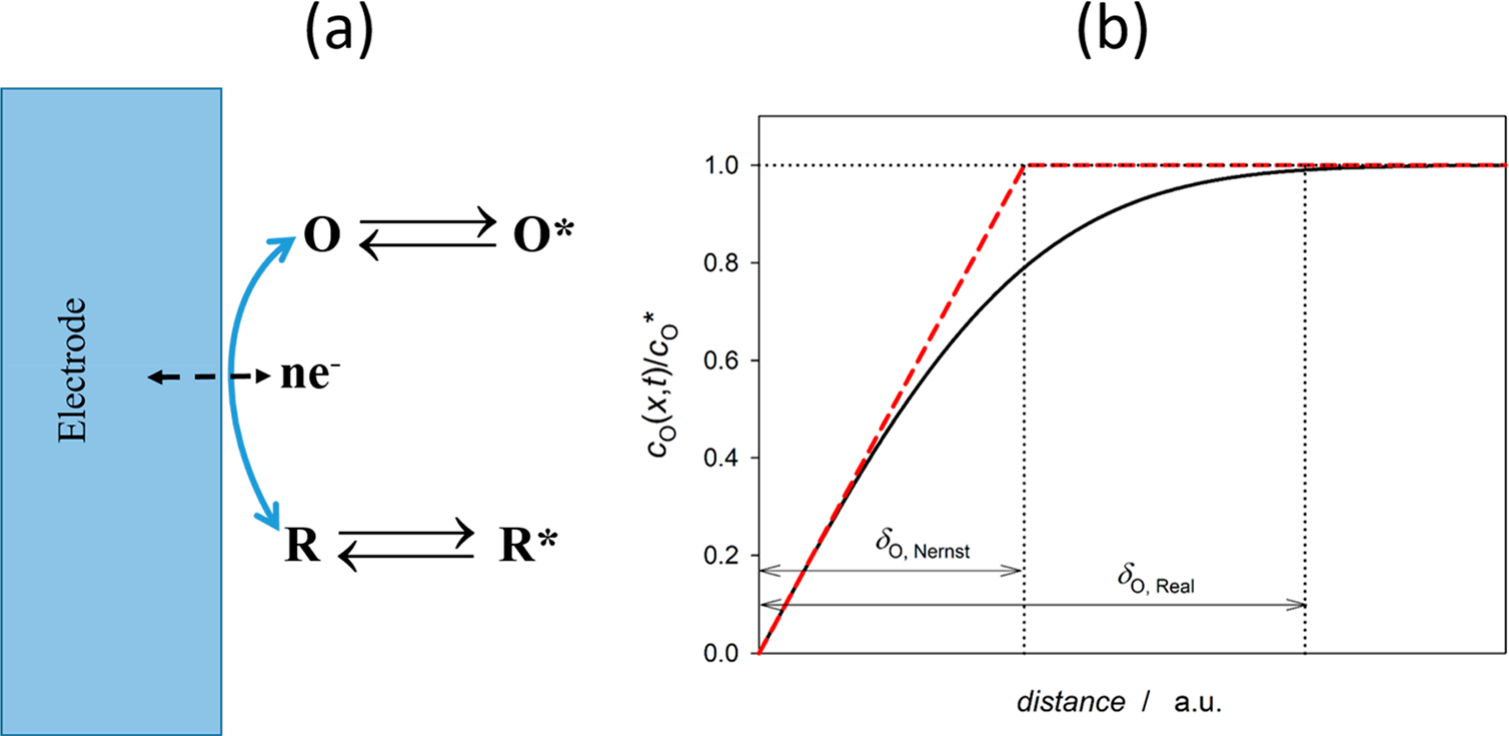
(a) Schematic of an electrode/solution interface where the charge transfer process  takes place. (b) Variation of the concentration of species O with the distance to the electrode surface (concentration profile), indicating the thicknesses of the linear and real diffusion layers (the latter can be determined for a certain maximum error, for example 1%, given the asymptotic behavior of the concentration profile) obtained upon the application of a constant potential *E* ≪ *E*^0^′ at a macroelectrode so that the surface concentration of species O is null. δ_O_ is the so-called Nernst linear diffusion layer for species O, which is defined as the distance from the electrode surface where the linear concentration profile (red dashed line) of such electroactive species reaches its bulk value.

Let us consider the following one-electron transfer process taking place at the electrode surface (Figure 2a)
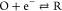
Iwith O being an oxidized species at an initial concentration *c*_O_^*^. When a potential difference is applied to the electrode–solution interface so that O is reduced to R, the concentration of O clearly decreases at the electrode surface, whereas species R is electrogenerated. Under these conditions, the equilibrium in the electrode vicinity breaks down and the transport of species O toward the electrode and of species R toward the bulk solution takes place.

If an inert (or supporting) electrolyte is added to the stagnant solution in a concentration at least 2 orders of magnitude higher than that of species O, apart from other important advantages such as the increase of the solution conductivity, it is possible to ensure that the transport of species O from the bulk solution to the electrode surface, and of species R in the opposite direction, occurs only by diffusion.^19,24,25^ The reduction of species O gives rise to an electric current that, based on Faraday’s and Fick’s first laws, is given by
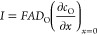
1with *D*_O_ (cm^2^/s) being the diffusion coefficient of species O, *A* (cm^2^) the electrode area, and *F* the Faraday constant (=96500 C/mol). The term (∂*c*_O_/∂*x*)_*x*=0_ in eq 1 refers to the concentration gradient of species O at the electrode surface that can be written in the following way:
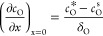
2where *c*_O_^s^ is the surface concentration of species O [Note: Although it is not possible to strictly define a “surface” or “interfacial” concentration, and surface excesses should be used instead, this term is employed here to refer to the concentration of electroactive species at the volumetric region next to the electrode interface. This fact has a direct implication in the dimensions of the electrochemical first-order rate constants, which are length/time.] and δ_O_ is defined in the caption of Figure 2. The expression for δ_O_ depends on the electrode geometry and on the mass transport mode, and among the electrodes considered, it is time-dependent in the case of macroelectrodes () [Note: Moreover, δ_O_ under nonsteady state conditions, that is, when it is dependent on time as in the case of macroelectrodes, is strictly dependent on the reversibility of the charge transfer reaction,^31^ although this fact has not been considered here.]; δ_O_ actually refers to a linear diffusion layer that is smaller than the perturbed zone in the vicinity of the electrode surface. In Table 1, the expressions corresponding to static planar, spherical, and disc electrodes under semi-infinite diffusion, and also to a rotating disc electrode (RDE) under convective-diffusion transport, are given.

**Table 1:**
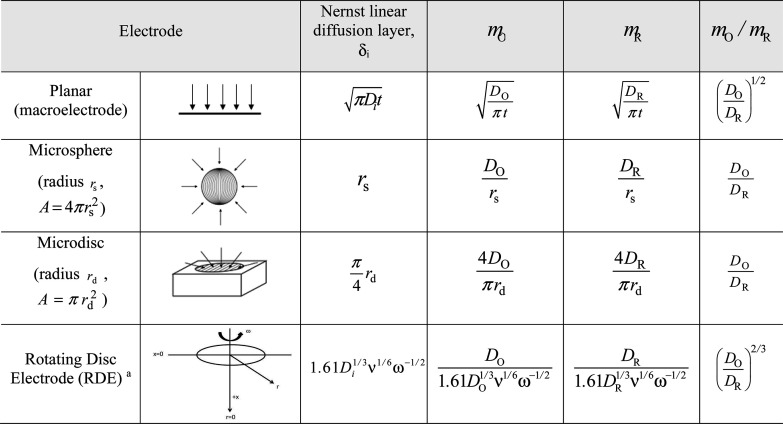
Expressions for the Nernst Linear Diffusion Layer, δ_i_, and the Mass Transport Coefficient, *m*, *m*_O_ for the Oxidized Species, and *m*_R_ for the Reduced One, for the Most Popular Electrode and Microelectrode Geometries under Semi-infinite Diffusion and for the RDE (Convective-Diffusion Mass Transport)

a Typically, the RDE consists of a disc electrode that is subject to a controlled rotational motion that provokes the motion of the fluid (electrolyte solution) near its surface as a function of the fluid kinematic viscosity, *v*, and of the speed of rotation, ω (rad/s).

It is important to highlight that at the electrode surface the sum of the fluxes of O and R must be null so that there is no accumulation of matter at the interface. This fact implies that
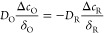
3with Δ*c*_i_ = (*c*_i_^*^ – *c*_i_^s^) where i ≡ O or R; thus, Δ*c*_R_ = −*c*_R_^s^ since it has been assumed that *c*_R_^*^ = 0.

Equation 1 can be rewritten as
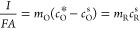
4where
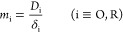
5is the mass transport coefficient. Under mass transport controlled conditions, which correspond to a null surface concentration of the oxidized species (i.e., *c*_O_^s^ = 0), eq 4 leads to
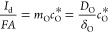
6with *I*_d_ being the mass transport limiting current for the reduction reaction.

### 2.a Reversible Electrochemical Reactions

If reaction I is very fast, one can consider that the surface concentrations of species O and R fulfill a relationship analogous to the Nernst equation:^19,24,25^
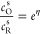
7with η being the dimensionless potential referred to the formal potential
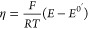
8where *E*^0^′ is the formal potential of the redox couple O/R. The combination of eq 4 and the following expressions of the concentrations of species O and R at the electrode surface results in:
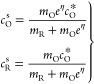
9

Thus, from eqs 2, 4, and 9, the dependence of the current with the applied potential (through the parameter η) is deduced:
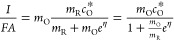
10from which the diffusion controlled current *I*_d_ defined in eq 6 can also be derived by making *E* → −∞ (*e*^η^ → 0). Note that, in the case of macroelectrodes, the current response is also dependent on time through the mass transfer coefficients (see Table 1), showing the typical Cottrellian dependence with *t*^–1/2^:
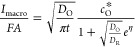
11

By combining eqs 6 and 10, the following relationship is obtained for the normalized current *I*/*I*_d_ of a reversible electrode reaction, which is independent of time, electrode size, or frequency of rotation:
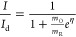
12

By solving for the potential in eq 12 (see also eq 8), it is deduced that (activity 1c in SI)
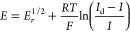
13where *E*_*r*_^1/2^ is the half-wave potential, defined as the potential at which *I* = *I*_d_/2 and given by (activity 1a in SI)
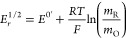
14so that, by plotting *E* vs ln((*I*_d_ – *I*)/*I*), the intercept gives direct access to the half-wave potential and the slope takes the value of 25.7 mV at *T* = 298 K, according to eqs 13 and 14. Note that the reversible half-wave potential is independent of time, but it depends on the kind of mass transport through the power of (*D*_R_/*D*_O_)^*x*^ (note that (*m*_R_/*m*_O_) = (*D*_R_/*D*_O_)^*x*^), which takes the value *x* = 1/2 for macroelectrodes, *x* = 1 for spherical or disc microelectrodes, and *x* = 2/3 for the RDE (see Table 1). In spite of this, a universal *I*/*I*_d_ vs *E* – *E*_*r*_^1/2^ relationship applies as shown in Figure 1c (see also problem 1b in SI).

Figure 1c shows the plot of the current–potential response deduced from eq 12 from which the reversible half-wave potential *E*_*r*_^1/2^ can be easily determined. It is also convenient to characterize the values of the potentials at which the current is three-quarters (*E*^3/4^) or one-quarter (*E*^1/4^) of the limiting current;^24^ thus, from eq 13 it is obtained that the difference |*E*_*r*_^3/4^ – *E*_*r*_^1/4^| for a reversible electrode reaction is given by (activity 2 in SI):
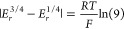
15which provides a simple criterion of reversibility, applicable whatever the electrode employed. It is worth highlighting that the above general expressions about the current–potential response (eqs 10 and 12), as well as its linearization as *E* vs ln((*I*_d_ – *I*)/*I*) (eq 13), hold for reversible electrode reactions regardless of the geometry and the dynamic or stagnant characteristics of the electrode considered. [Note: This is due to the current–potential response of reversible electron transfers being *rigorously* given by the product of a potential-dependent factor (*c*_O_^*^ – *c*_O_^s^) or *c*_R_^s^ in eq 4 and a potential-independent factor (*m*_O_ or *m*_R_ in eq 4).^19^]

### 2.b Nonreversible Electrochemical Reactions

For an electron transfer reaction of slow kinetics, the surface condition given by eq 7 is to be replaced by a Butler–Volmer-like equation:^27^
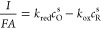
16where
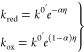
17with *k*^0^′ being the standard heterogeneous rate constant (typically in cm s^–1^), and α and (1 – α) are the charge transfer coefficients for the cathodic and anodic reactions, respectively, with 0 < α < 1, usually taking values close to 0.5.^17−20^

From eqs 4 and 6, the following expressions for *c*_O_^s^ and *c*_R_^s^ are obtained:
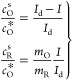
18

By combining eqs 6, 16, and 18, the normalized current can be written as
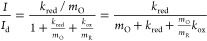
19and the surface concentrations are given by
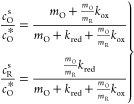
20

Note that, unlike the case of reversible processes, the response is affected by the electrode characteristics (either macrometric or micrometric, either static or rotating) through the mass transfer coefficients. An Excel spreadsheet is provided as Supporting Information for the calculation of the current–potential response (eq 19) and the surface concentrations–potential curves (eq 20) for all the electrodes considered and any degree of reversibility.

**Figure 3:**
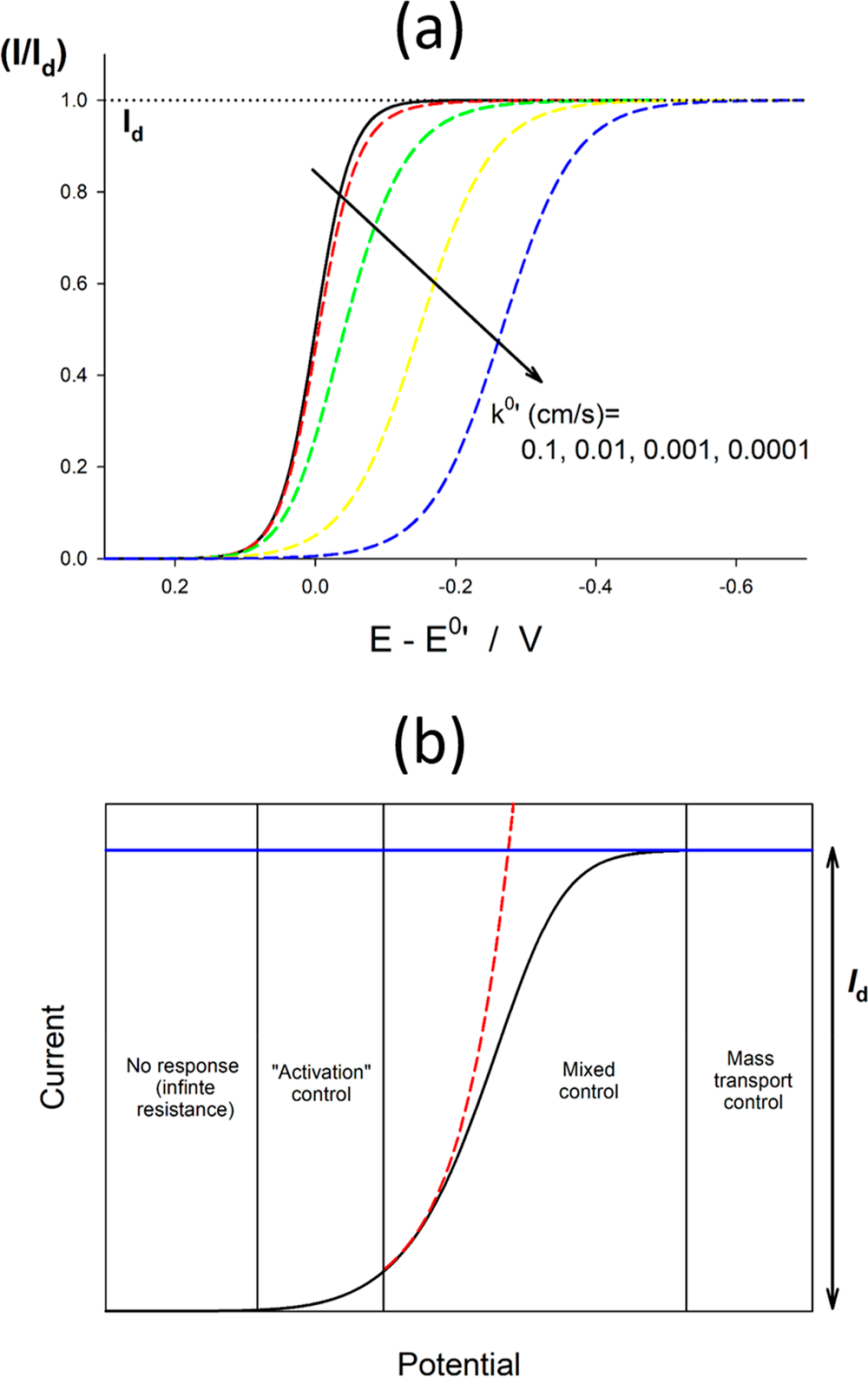
(a) Semidimensionless current–potential response of nonreversible (dashed line) electrode reactions as a function of the value of *k*^0^′, with α = 0.5, *m*_O_ = *m*_R_ = 0.018 cm/s (eq 19), and *T* = 298 K. Black solid line: reversible process *k*^0^′ ≥ 1 cm/s. (b) Schematic of the current–potential response of a nonreversible electrode reaction, indicating the different control regimes as a function of the applied potential.

Figure 3a shows the *I*/*I*_d_ – *E* curves obtained from eq 19 for different values of *k*^0^′ and α = 0.5. As can be observed, the decrease of the value of *k*^0^′ gives rise to the shift of the wave toward more negative potentials; that is, the reduction of O to R is hindered. For the nonreversible cases, three different zones of the *I*/*E* curve can be distinguished (see Figure 3b): the foot of the wave, where the diffusive mass transport is not effective and the current is only controlled by the kinetics of the charge transfer process (activation control); potentials around the half-wave potential *E*_irrev_^1/2^ (see below), where a diffusive-kinetic mixed control takes place; and, finally, potentials notably more negative than *E*_irrev_^1/2^ corresponding to the region of mass transport.

### 2.c Reversible Limit

As illustrated in Figure 3a, under typical mass transport conditions, an electrode process behaves as reversible (black line in Figure 3a) for values of *k*^0^′ ≥ 1 cm/s. Indeed, under these conditions, by making *k*_red_ → ∞ and *k*_ox_ → ∞ in eq 19, the expression for reversible processes (eq 12) is obtained.

### 2.d Fully Irreversible Limit

In the opposite case, *k*^0^′ ≪ 1 cm s^–1^, reaction I behaves as totally irreversible; that is, it occurs at very negative potentials for which *k*_red_ ≫*k*_ox_. Note that this condition can hold for *k*^0^′ values corresponding to quasi-reversible reactions as long as *E* ≪ *E*^0^′, that is, by selecting negative enough potential values for the study, corresponding to the top of the *I–E* curve where the process behaves as fully irreversible. By taking this into account in eq 19, it can be deduced that
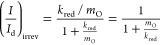
21

This equation holds for the curves with *k*^0^′ < 10^–3^ cm/s in Figure 3a. In this case the half-wave potential can be easily obtained by making *I* = *I*_d_/2 in eq 21 (see also eq 17 ; activity 4a in SI)
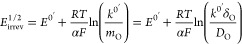
22

Note that the *E*_irrev_^1/2^ value depends on the kinetic constant *k*^0^′ and on the diffusion layer through *m*_O_ (=*D*_O_/δ_O_). Hence, unlike the case of a reversible process, the diffusion layer plays a key role and the electrode characteristics have an important influence on *E*_irrev_^1/2^ so that its value depends on time at a macroelectrode (, see Table 1), on the electrode geometry for microelectrodes, or on the rotation rate in the case of RDEs. This behavior of *E*_irrev_^1/2^, and so of the position of the *I–E* wave, can be used as a diagnostic criterion about the reversibility of the electrode reaction. Thus, *E*_irrev_^1/2^ shifts toward more cathodic potentials as *k*^0^′ decreases and also as the diffusion layer thickness is smaller, that is, for shorter pulses at macroelectrodes, ultramicroelectrodes of smaller radius, and faster rotation rates at RDEs; note that these variables do not affect the position of the current–potential wave of reversible processes (eq 14). Thus, the apparent irreversibility of an electrode reaction is strongly dependent on the experimental time scale and on the electrode employed.

**Figure 4:**
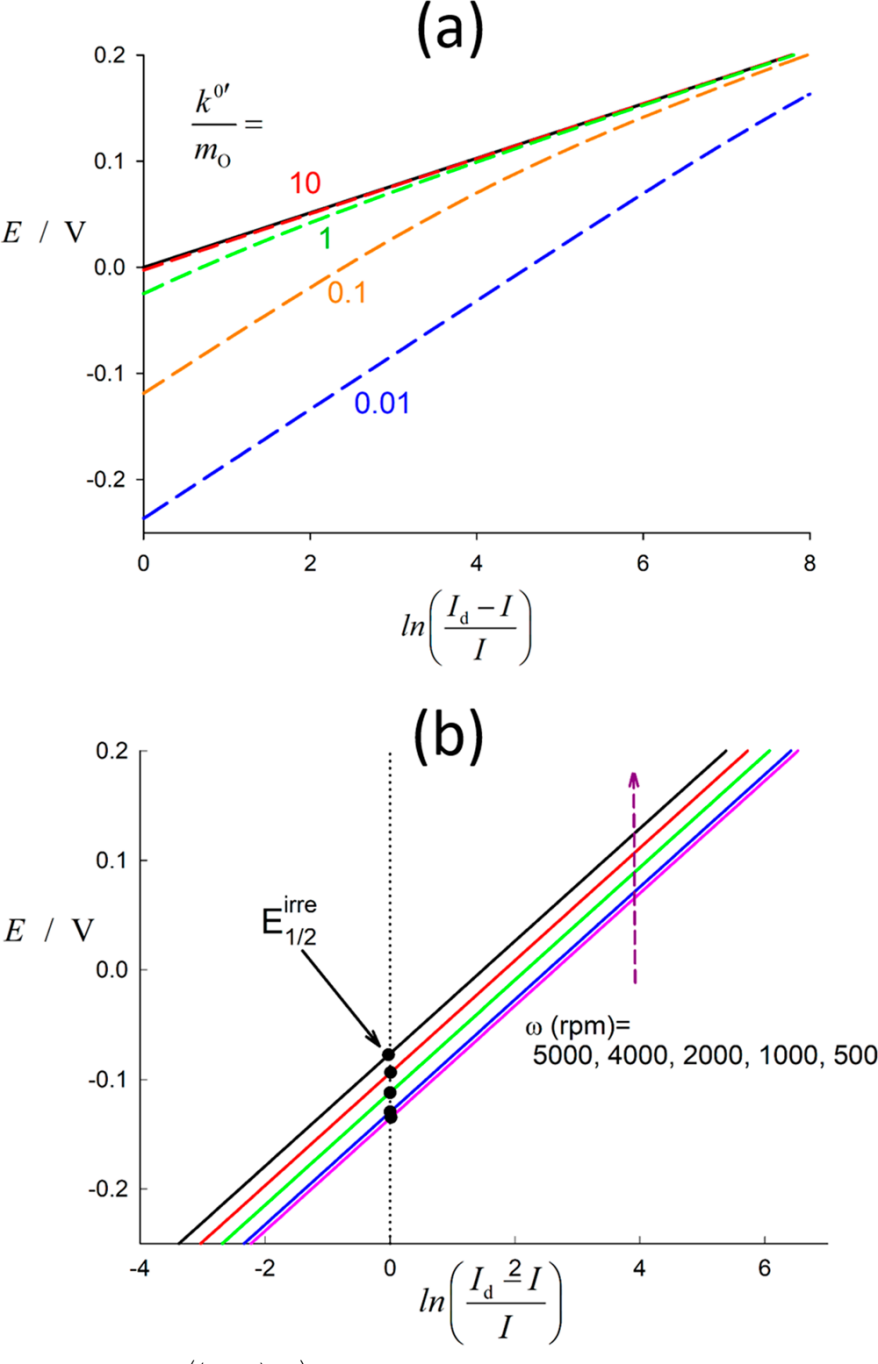
(a) *E* vs ln((*I*_d_ – *I*)/*I*) plot as a function of the reversibility of the electrode reaction parametrized through the value of the ratio *k*^0^′/*m*_O_, with α = 0.5, *m*_R_ = *m*_O_. (b) *E* vs ln((*I*_d_ – *I*)/*I*) plot of a fully irreversible electrode reaction (*k*^0^′ = 10^–3^ cm/s, α = 0.5, *D* = 10^–5^ cm^2^/s, ν = 1.09 × 10^–2^ cm^2^/s) at an RDE as a function of the angular frequency of rotation.

**Figure 5:**
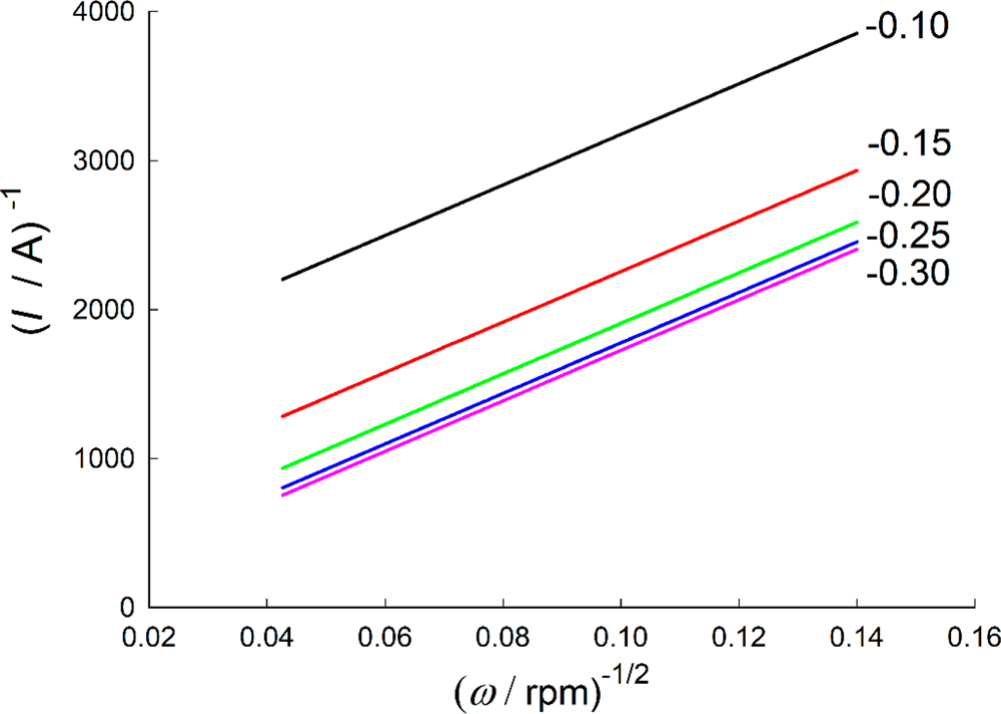
Variation of the inverse of the current density with ω^–1/2^ for an irreversible electrode reaction at an RDE. *k*^0^′ = 10^–3^ cm/s, α = 0.5. The values of *E* – *E*^0^′ applied (in V) are indicated on the curves.

It is also possible to write eq 21 in two different ways that are more appropriate when it comes to extracting the characteristic parameters of process I. First, analogously to a reversible process (eq 13), the *I–E* response given by eq 21 can be linearized by plotting *E* vs ln((*I*_d_ – *I*)/*I*) [Note: In the case of quasi-reversible processes, deviations from linearity are expected for the *E* vs ln ((*I*_d_ – *I*)/*I*) plot^18^ (see Figure 5a), and the current–potential response is to be analyzed with the complete eq 19.], and the half-wave potential, *E*_irrev_^1/2^, can be determined from the corresponding intercept (see Figure 4a and activity 4d in SI)
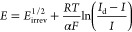
23which allows us to calculate the value of *k*^0^′, once *m*_O_ and *E*^0^′ are known (see eq 22). With regard to the slope of the plot *E* vs ln((*I*_d_ – *I*)/*I*) of irreversible reactions, according to eq 23, this is larger than that of reversible reactions (i.e., >25.7 mV), independent of the electrode employed, and it enables us to extract the value of the transfer coefficient, α.

From eq 23, it is readily obtained that the difference |*E*_*irrev*_^3/4^ – *E*_*irrev*_^1/4^| for an irreversible electrode reaction is given by (activity 5 in SI):
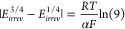
24

From the comparison of eqs 15 and 24, taking into account that the α-value is smaller than unity (typically around 0.5), it is concluded that the difference |*E*^3/4^ – *E*^1/4^| is larger for irreversible electrode reactions than for reversible processes, serving as a simple kinetic diagnosis criterion and enabling the determination of α in the case of irreversible electron transfers.^24^

Equation 21 can be also written in an inverse form as
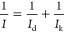
25with *I*_k_ being the kinetic current in the absence of mass transport
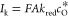
26

Thus, in general, eq 25 can be written as
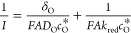
27and specifically for RDEs as (activity 6 in SI)
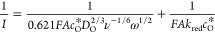
28so that 1/*I* at a given potential varies linearly with δ_O_ (specifically with ω^–1/2^ for RDEs), with a slope depending on the diffusion coefficient of the reactant and an intercept that allows for the determination of *k*_red_ (and *k*^0^′). Attending to the expressions given in Table 1, the value of δ_O_ can be varied easily at macroelectrodes (by using different pulse times) and at the RDE (by employing different rotation rates); in the case of microelectrodes, the study is in general more laborious since it involves using electrodes of different sizes. As an example, Figure 5 shows the curves 1/*I* vs δ_O_ (1/*I* vs ω^–1/2^ for a RDE) for a value of *k*^0^′ = 10^–3^ cm/s and different values of the applied potential, *E* – *E*^0^′.

Note that eqs 21, 23, and 27 are indeed the same expression, written in three different ways, for the current–potential response of a totally irreversible charge transfer process. These different ways of treating the current and potential data are related with different experimental protocols for obtaining kinetic information.

### 2.e Tafel Analysis

The well-known Tafel analysis is due to Julius Tafel, and it is based on a linearization of the current–potential response under conditions of redox kinetic control. Thus, from eqs 6 and 21, it can be easily deduced
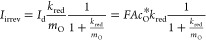
29

At the foot of the current–potential wave, the influence of the mass transport is not relevant (activation or kinetic control region in Figure 3b) and, by making *k*_red_ ≪ *m*_O_ in eq 29, the response simplifies to
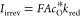
30

Note that the applicability of the Tafel analysis is more limited than that of the Koutecký–Levich one since the simultaneous fulfilment of the conditions *k*_red_ ≫ *k*_ox_ and *k*_red_ ≪ *m*_O_ is only possible at the foot of the wave of processes with a very small *k*^0^′ value; in other words, the Tafel analysis is not accurate for the study of quasi-reversible reactions.

Taking the decimal logarithm on both sides of eq 30, and taking into account the expression of *k*_red_ given by eq 17, the following is obtained
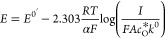
31

Under these conditions, the plot of *E* vs log(*I*) should yield a straight line (with a slope of around 118 mV/dec for α = 0.5 and *T* = 298 K).

## 3 Implementation

The theoretical framework was introduced to 15 upper-level undergraduate students in an advanced course on Physical Chemistry for a Chemistry degree. The time schedule for the development of the complete module was 5 h, combining both theoretical classes and activities. The information about the students’ relevant background was anecdotal, i.e., no pretest was done in order to measure this background or the lack of it, but evidence was acquired through interviews prior to carrying out the module. In this sense, the students indicated that they had a basic background in theoretical topics related to electrode kinetics or mass transport. Most of them had little knowledge relative to the Butler–Volmer equation, and they clearly ignored its range of validity (i.e., the influence of mass transport). The majority of topics mentioned by the students related to Electrochemistry were restricted to equilibrium (Nernst equation) and ionic transport phenomena, although all of them indicated that they had previously studied some notions relative to electrostatics. Nevertheless, all of the students claimed that they were interested in different questions about practical aspects of electrochemical devices, such as fuel cells or batteries, and that they had some limited experience in basic electrochemical techniques through the different laboratory activities during their Chemistry degree.

The intended learning outcome of the approach followed here is that the students become able to achieve, in a rational and easy way, an understanding of the concepts of the half-wave potential and the limiting current, the dependence of the former on the kinetics of the process and the characteristics of the mass transport conditions, as well as the determination of relevant kinetic parameters from the analysis of the current–potential response. For this, the in-class developments of the theoretical topics were combined with exercises by using the Excel sheet, especially for the cases of fully irreversible reactions. A short training in the simulation spreadsheet and in the meaning of the different parameters was carried out before the students addressed the activities. The exercises proposed were solved by the students in an activity report sheet (see Section S2 in Supporting Information), which also included some questions relative to the manipulation of the theoretical expressions. As a complement to this short course, some bibliographic examples of the electrochemical conversion of small molecules, such as hydrogen or oxygen, in fuel cells and metal–air batteries could be discussed (see refs (28−30)). The follow-up discussion would be focused on the characterization of the performance of the electrochemical reactions taking place, and, briefly, on the importance of the catalysis.

## 4 Student Learning Assessment

**Figure 6:**
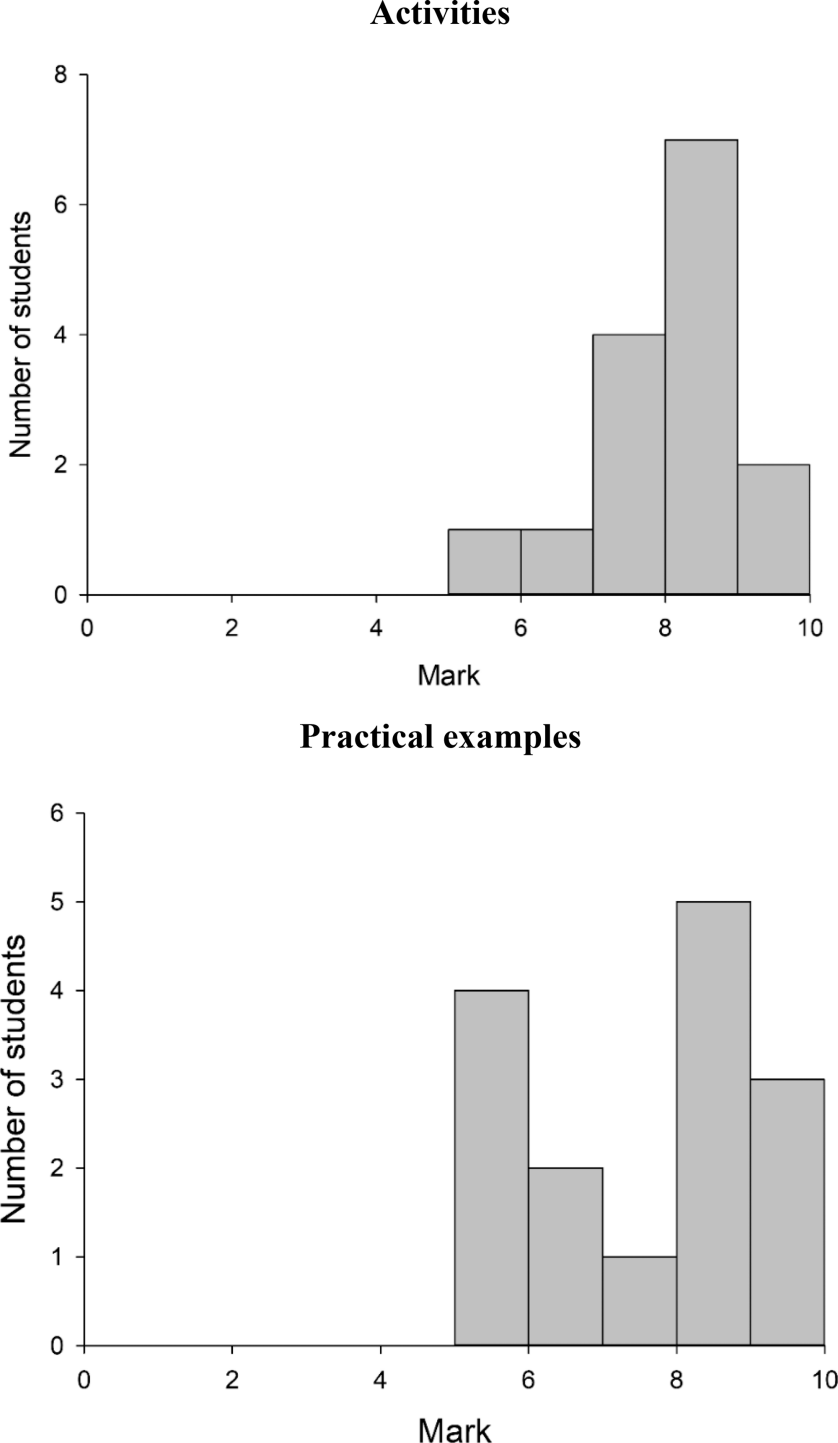
Performance of 15 upper-level undergraduate students in the activities and practical examples proposed in Section S2 of the Supporting Information according to the marking guides given in Section S3 of the Supporting Information.

Different strategies were followed in order to collect data about the students’ performance in the proposed activities and practical examples. The 15 activity report sheets delivered by the students were analyzed on the basis of marking guides specifically designed (see Section S3 in Supporting Information). Figure 6 shows the overall results obtained, with all the students meeting the minimum passing score of 5 in both activities and practical examples. The overall performance in the former was better than in the latter, mainly due to some of the students completing the kinetic analysis, but finding difficulty in the critical discussion of their results. Additionally, the students’ performance was followed by direct observation together with a discussion with them about the main problems they found (both during the activities, as well as after finishing the whole module). On the basis of the collected results, the following conclusions can be drawn:

- Most of the students find difficulties in the comprehension of interfacial processes, which are the basis of the electrochemical behavior under study. The students indicate that mostly homogeneous processes were analyzed during their degree. In this sense, they claim that the introduction of mass transport coefficients was helpful for them, especially for comprehending the differences between the mass transport modalities arising from the electrode geometric characteristics or from the presence of convection. Such crucial notions should pave the way for the sound understanding and applications of more complex and widely used transient techniques, such as cyclic voltammetry.
- The transient nature of the electrochemical responses is an additional obstacle in the students’ learning experience. It is expected that the activities proposed and the theoretical discussion focused on mass transport coefficients and limiting currents (eqs 4–6) will be helpful and should improve their understanding of the factors defining the overall response, especially for nonreversible situations.
- The students claim that the theoretical framework had helped them in noticing and rationalizing the interplay between the charge transfer kinetics and the mass transport. They also indicate that the manipulation of the Excel sheet was a simple task for them, and that the differences observed depending on the mass transport mode considered were striking, especially for the case of irreversible processes. In this case, the simple mathematical expressions employed allow them to focus on the physical meaning of the different parameters under study.
- The students were able to solve the different activities proposed in the report sheet. Even so, they found some difficulties in identifying and manipulating the appropriate mathematical expressions for each of the situations under study, as well as in the selection and conversion of magnitude units. On the other hand, the students managed well and straightforwardly with running the simulations of the different cases with the Excel sheet. This is a very important result since it reflects how the students can struggle with the physicochemical fundamentals and on paper mathematical derivations of the problem, but this does not prevent them from the (inappropriate) use of user-friendly software.
- The adequate selection of a given plot for obtaining relevant data (rate constants, half-wave potentials, ...) is not a trivial task for the students. The limit of applicability of well-known protocols, such as the Tafel plot, is unknown for them, and it is typical that they apply these protocols even when they are not adequate, therefore obtaining physically meaningless results. This is a point that needs to be clearly specified in the teaching of Electrochemistry in order to avoid misinterpretations.

## 5 Conclusions

Understanding the rate of heterogeneous electron transfer processes at an interface, as influenced by the interplay between the redox kinetics and the species mass transport, is crucial for the rational development of uprising electrochemical-based technologies and materials. The main goal of the framework proposed here is to help the students to achieve an adequate understanding of the physical meaning and implications of the heterogeneous nature of the electrochemical phenomena. This is not a simple task since heterogeneity in chemical systems is almost practically absent in the Chemistry graduate curriculum, and the interfacial nature of electrochemical phenomena is a cornerstone in the understanding of these reactions. Through the theoretical treatments and practical developments presented here, this topic can be introduced in a simple yet accurate way in order to provide the students with a solid background to comprehend, predict, and interpret the voltammetric response of electrode reactions under different and most frequent experimental conditions. Thus, for reversible (fast) reactions, the half-wave potential is dependent on the kind of mass transport (linear diffusion, radial diffusion, convective-diffusion, ...). In the case of nonreversible processes, the apparent degree of reversibility is the result of the electron transfer kinetics *relative to* the rate of mass transport. Hence, the voltammetric wave depends on the ratio between the standard heterogeneous rate constant and the mass transfer coefficient (*k*^0^′/*m*). This leads to that the *E*_1/2_ value depends on time in the case of macroelectrodes, on the electrode dimension at microelectrodes, or on the rotation rate at rotating disc electrodes.

In the two limit kinetic regimes of reversible and fully irreversible electrode reactions, the linearization of the current–potential curves is possible, and it enables the application of simple protocols for the determination of thermodynamic and kinetic parameters, which are sometimes misapplied in the study of intermediate kinetics (quasi-reversible reactions). In such situations, the simple mathematical expressions presented here enable the accurate analysis of experimental current–potential responses.

The students’ performance in solving the different activities proposed here reveals that these concepts can be introduced in the Chemistry graduate curriculum in a simple and direct way with satisfying results. Nevertheless, it is important to consider this framework as a part of a more general theoretical–experimental approach to these reactions, which should be combined, if possible, with laboratory examples of well-known systems in order to achieve adequate learning of these relevant topics. These are cornerstones for the students to cope with more elaborate techniques, such as cyclic voltammetry, in a solid and rational way.

## Supporting Information Available

Excel calculator of the current–potential (eq 19) and the surface concentration–potential (eq 20) curves (ZIP)
Glossary of terms, activities and practical examples for students, and marking guides for the activities and practical examples (PDF, DOCX)
