# Supplementary material for: Voltammetric Kinetic Studies of Electrode Reactions: Guidelines for Detailed Understanding of Their Fundamentals
Source: J Chem Educ. 2022 Dec 27;100(2):697–706. doi: 10.1021/acs.jchemed.2c00944 (PMC9933535; doi:10.1021/acs.jchemed.2c00944)
Supplement: Supplementary file 2 — ed2c00944_si_002.pdf [file ed2c00944_si_002.pdf]

SUPPORTING INFORMATION

Voltammetric kinetic studies of electrode reactions: Guidelines for detailed understanding of their fundamentals

Joaquín González, Eduardo Laborda, Ángela Molina*

Departamento de Química Física, Facultad de Química, Regional Campus of International Excellence "Campus Mare Nostrum", Universidad de Murcia, 30100 Murcia, Spain

* Email: amolina@um.es

Content

S1. GLOSSARY

S2. ACTIVITIES FOR STUDENTS

S3. MARKING GUIDES

S4. BIBLIOGRAPHY

S1. GLOSSARY

Symbol	Meaning	Usual units
α	Transfer coefficient for reduction	none
$1-\alpha$	Transfer coefficient for oxidation	none
A	Electrode surface area	cm^2
$c_i(x,t)$	Concentration profiles of species i (≡ O, R)	mol/cm^3
c_i^s	Surface concentration of species i (≡ O, R)	mol/cm^3
c_O^*	Bulk concentration of the oxidized species O	mol/cm^3
D_i	Diffusion coefficient of species i (≡ O, R)	cm^2/s
δ_i	Thickness of the linear diffusion layer for species i (≡ O, R)	cm
E	Applied potential at the working electrode versus a reference electrode	V
$E^{0'}$	Formal potential of the redox couple O/R	V
$E_r^{1/2}$	Half-wave potential of reversible (fast) electrochemical reactions	V
$E_{irrev}^{1/2}$	Half-wave potential of irreversible electrochemical reactions	V
$E^{3/4}$	Potential at which the current is 3/4 of the limiting current	V
$E^{1/4}$	Potential at which the current is 1/4 of the limiting current	V
η	Dimensionless potential referred to the formal potential	none
I	Electric current	A
I_d	Mass transport-limited current	A
I_k	Kinetic current in the absence of mass transport	A
$k^{0'}$	Standard heterogeneous rate constant	cm/s
k_{red}	Heterogeneous rate constant for reduction	cm/s
k_{ox}	Heterogeneous rate constant for oxidation	cm/s
m_i	Mass transport coefficient for species i (≡ O, R)	cm/s
t	Time of the applied potential perturbation	s
T	Absolute temperature	K
x	Distance to the electrode surface	cm

S2. ACTIVITIES FOR STUDENTS

Reversible electron transfers

1. From Eqn. (12) of the main manuscript:

a) Derive the expression of the half-wave potential of reversible electrode reactions (Eqn. (14)).

b) Derive the following expression for the current where the half-wave potential is taken as reference (instead of the formal potential):

$$\frac{I}{I_{\mathrm{d}}} = \frac{1}{1 + e^{\eta_{1/2,rev}}}$$

with:

$$\eta_{1/2,rev} = \frac{F}{RT}\left(E - E_r^{1/2}\right)$$

c) Derive the expression for the linear plot E *vs* $ln\left(\frac{I_{\mathrm{d}} - I}{I}\right)$ (Eqn. (13)).

d) Deduce the value of the cathodic ($E \to -\infty$) and anodic limiting current ($E \to +\infty$) and justify the results obtained.

Hint: Note that in the derivation of Eqn. (12) it has been assumed that only species O is present in solution.

2. From Eqn. (13), derive the following expressions for the potentials at which the current value is one quarter ($I = \frac{1}{4} I_{\mathrm{d}}$) or three quarters ($I = \frac{3}{4} I_{\mathrm{d}}$) of the limiting current:

$$E_r^{1/4} = E_r^{1/2} + \frac{RT}{F} ln(3)$$

$$E_r^{3/4} = E_r^{1/2} - \frac{RT}{F} ln(3)$$

from which the expression for the difference between them (Eqn. (15)) is obtained.

Non-reversible electron transfers

3. From the general solution for the current-potential response of electrode processes of any reversibility (Eqn. (19)):

a) Derive the expression corresponding to reversible processes (Eqn. (12)) as the limit when $k_{\mathrm{red}} \to \infty$ and $k_{\mathrm{ox}} \to \infty$.

b) Derive the expressions for the cathodic and anodic limiting currents and compare the results with those obtained for a reversible electrode reaction.

4. From Eqn. (21) for fully irreversible electrode processes:

a) Derive the expression of the half-wave potential of irreversible electro-reduction reactions (Eqn. (22)).

b) Taking into account that the exact expression for $E_{\mathrm{irrev}}^{1/2}$ at macroelectrodes is given by

$$E_{\mathrm{irrev}}^{1/2} = E^{0'} + \frac{RT}{\alpha F}\ln\left(2.309k^{0'}\sqrt{\frac{t}{D_{\mathrm{O}}}}\right)$$

determine the absolute error of the approximate expression (22).

c) Derive the following expression for the current where the half-wave potential is taken as reference (instead of the formal potential):

$$\left(\frac{I}{I_{\mathrm{d}}}\right)_{\mathrm{irrev}} = \frac{1}{1+e^{\alpha\eta_{1/2,irrev}}}$$

with:

$$\eta_{1/2,irrev} = \frac{F}{RT}\left(E - E_{irrev}^{1/2}\right)$$

d) Derive the linearized plot E *vs* $ln\left(\frac{I_{\mathrm{d}}-I}{I}\right)$ (Eqn. (23)).

5. From Eqn. (23), for an irreversible process, derive the following expressions for the potentials at which the current value is one quarter ($I = \frac{1}{4}I_{\mathrm{d}}$) or three quarters ($I = \frac{3}{4}I_{\mathrm{d}}$) of the limiting current:

$$E_{irrev}^{1/4} = E_{irrev}^{1/2} + \frac{RT}{\alpha F}ln(3)$$

$$E_{irrev}^{3/4} = E_{irrev}^{1/2} - \frac{RT}{\alpha F} ln\left(3\right)$$

from which the expression for the difference between them (Eqn. (24)) is obtained.

6. From Eqn. (21), deduce the Koutecký-Levich equation for irreversible reactions at rotating disc electrodes (Eqn. (28)).

Practical examples

1. In the Excel file "SI - Practical example 1.xlsm", the theoretical I (A) *vs* E (V) response is calculated for the irreversible reduction of Fe(III) on Pt, Fe(III) + e$^-$ → Fe(II), for which $E^{0'}$ = 0.771 V (vs SHE) and considering $D_{\mathrm{Ox}} = D_{\mathrm{Red}} = 10^{-5}$ cm^2/s, $c_{\mathrm{O}}^{*} = 1$ mM and T = 298 K. With the I (A) *vs* E (V) data corresponding the case of a planar electrode with A = 0.4 cm^2 and $t = 1$ s, perform the following kinetic analyses:

a) Verify the linear relationship between E(V) and $ln\left(\frac{I_{\mathrm{d}} - I}{I}\right)$. On the basis of Eqn. (23), obtain the values of the transfer coefficient, α, and of the standard heterogeneous rate constant, $k^{0'}$, via simple linear regression analysis.

b) Prepare the Tafel plot E *vs* $\log(I)$ at potentials corresponding to the foot of the wave (approximately 15% of the maximum current). On the basis of Eqn. (31), determine the values of α and $k^{0'}$ via simple linear regression analysis of the data.

c) Compare your results with the values reported for the kinetic parameters of this system: $k^{0'} \approx 9 \times 10^{-6}$ cm/s (25ºC) and $\alpha = 0.50$ [1].

2. In the Excel file "SI - Practical example 2.xlsm", the theoretical I (A) *vs* E (V) response is calculated for the irreversible reduction of Eu(III) on Hg, Eu(III) + e$^-$ → Eu(II), for which $E^{0'}$ = –0.160 V (vs SHE) and considering $D_{\mathrm{Ox}} = D_{\mathrm{Red}} = 10^{-5}$ cm^2/s, $c_{\mathrm{O}}^{*} = 1$ mM and T = 298 K. With the I (A) *vs* E (V) data for the following cases:

- Macroelectrode with A = 0.8 cm^2 and $t = 1$ s.
- Disc ultramicroelectrode with $r_{\mathrm{d}} = 5$ μm.
- Rotating disc electrode (RDE) with A = 0.8 cm^2, $\omega = 100$ rad/s and $\nu = 0.0091$ cm^2/s (water).

perform the following kinetic analyses:

a) For all the above electrodes, verify the linear relationship between $E(\mathrm{V})$ and $ln\left(\frac{I_{\mathrm{d}}-I}{I}\right)$, and obtain the values of the half-wave potential. In each case, on the basis of Eqn. (23), obtain the values of the transfer coefficient, α, and of the standard heterogeneous rate constant, $k^{0'}$, via simple linear regression analysis.

b) Justify the shift of the half-wave potential towards more negative values as the electrode size shrinks.

c) According to Eqn. (28), prepare a Koutecký-Levich plot, $1/I$ *vs* $1/\omega^{1/2}$, for the RDE (for example, in the range $10 < \omega(\mathrm{rad/s}) < 500$) at four different E-values: –0.250, –0.275, –0.300 and –0.325 V. For each E–value, obtain the corresponding k_{red}–value and then carry out the simple linear regression analysis of $\ln(k_{\mathrm{red}})$ *vs* E to determine the values of α and $k^{0'}$.

d) In all cases, compare your results with the values of the kinetic parameters of this system: $k^{0'}$ = (1.4–1.7) x 10^{-4} cm/s (25ºC), α = 0.66 – 0.69 [2].

S3. MARKING GUIDES

S3.1. Activities

	Criteria	Weight
**Organization and presentation**	• Solution is written in a clear and legible way.	10%
**Mathematical manipulations**	• All necessary information is identified correctly.<br>• Mathematical derivations are correct.<br>• Progression from the first to the last steps is detailed.	30%
**Variables and constants**	• Physical magnitudes and constants are identified and their symbols are correct.	15%
**Results**	• The final expression is correct.	25%
**Analysis of results**	• Most relevant findings and conclusions are identified.<br>• There is evidence of critical thought process.<br>• Results are compared with those in bibliography.	20%

S3.2. Practical examples

	Criteria	Weight
**Organization and presentation**	• Report is written in a clear and legible way.	10%
**Mathematical manipulations**	• All necessary information is identified correctly.<br>• Mathematical derivations are correct.<br>• Progression from the first to the last steps is detailed.	20%
**Data analysis**	• Conditions of applicability of the equations are identified.<br>• Graph axes are labelled and scaled appropriately.<br>• Simple linear regression is performed and reported correctly.	25%
**Variables, constants and units**	• Physical magnitudes and constants are identified and their symbols are correct.<br>• Adequate units are indicated in the text and graphs.	15%
**Results**	• The final expression or value is correct.	15%
**Analysis of results**	• Most relevant findings and conclusions are identified.<br>• There is evidence of critical thought process.<br>• Results are compared with those in bibliography.	15%

S4. BIBLIOGRAPHY

(1) Oldham, K. B.; Myland, J. C.; Bond, A. M. *Electrochemical Science and Technology: Fundamentals and Applications*; John Wiley & Sons: Chichester, 2012.

(2) Henstridge, M. C.; Laborda, E.; Wang, Y.; Suwatchara, D.; Rees, N.; Molina, A.; Martínez-Ortiz, F.; Compton, R. G. Giving Physical Insight into the Butler–Volmer Model of Electrode Kinetics: Application of Asymmetric Marcus–Hush Theory to the Study of the Electroreductions of 2-Methyl-2-Nitropropane, Cyclooctatetraene and Europium(III) on Mercury Microelectrodes. *J. Electroanal. Chem.* **2012**, *672*, 45–52. https://doi.org/10.1016/j.jelechem.2012.02.028.
