# Supplementary material for: Voltammetric Kinetic Studies of Electrode Reactions: Guidelines for Detailed Understanding of Their Fundamentals
Source: J Chem Educ. 2022 Dec 27;100(2):697–706. doi: 10.1021/acs.jchemed.2c00944 (PMC9933535; doi:10.1021/acs.jchemed.2c00944)
Supplement: Supplementary file 3 — ed2c00944_si_003.docx [file ed2c00944_si_003.docx]

**SUPPORTING INFORMATION**

**Voltammetric kinetic studies
of electrode reactions: Guidelines for detailed understanding of their fundamentals**

**Joaquín González, Eduardo Laborda, Ángela Molina^*^**

*Departamento de Química Física, Facultad de Química, Regional Campus of International Excellence “Campus Mare Nostrum”, Universidad de Murcia, 30100 Murcia, Spain*

* Email: amolina@um.es

**Content**

S1. GLOSSARY

S2. ACTIVITIES FOR STUDENTS

S3. MARKING GUIDES

S4. BIBLIOGRAPHY

**S1. GLOSSARY**

| **Symbol** | **Meaning** | **Usual units** |
| --- | --- | --- |
| α | Transfer coefficient for reduction | none |
| 1 − α | Transfer coefficient for oxidation | none |
| *A* | Electrode surface area | cm^2^ |
| $c_{\text{i}}\text{(}x,t\text{)}$ | Concentration profiles of species i (≡ O, R) | mol/cm^3^ |
| $c_{\text{i}}^{\text{s}}$ | Surface concentration of species i (≡ O, R) | mol/cm^3^ |
| $c_{\text{O}}^{*}$ | Bulk concentration of the oxidized species O | mol/cm^3^ |
| *D*_i_ | Diffusion coefficient of species i (≡ O, R) | cm^2^/s |
| $\delta_{\text{i}}$ | Thickness of the linear diffusion layer for species i (≡ O, R) | cm |
| $E$ | Applied potential at the working electrode  versus a reference electrode | V |
|  | Formal potential of the redox couple O/R | V |
| $E_{r}^{1/2}$ | Half-wave potential of reversible (fast) electrochemical reactions | V |
| $E_{irrev}^{1/2}$ | Half-wave potential of irreversible electrochemical reactions | V |
| $E^{3/4}$ | Potential at which the current is 3/4 of the limiting current | V |
| $E^{1/4}$ | Potential at which the current is 1/4 of the limiting current | V |
| $\eta$ | Dimensionless potential referred to the formal potential | none |
| $I$ | Electric current | A |
| $I_{\text{d}}$ | Mass transport-limited current | A |
| $I_{\text{k}}$ | Kinetic current in the absence of mass transport | A |
| $k^{0'}$ | Standard heterogeneous rate constant | cm/s |
| $k_{\text{red}}$ | Heterogeneous rate constant for reduction | cm/s |
| $k_{\text{ox}}$ | Heterogeneous rate constant for oxidation | cm/s |
| *m*_i_ | Mass transport coefficient for species i (≡ O, R) | cm/s |
| *t* | Time of the applied potential perturbation | s |
| *T* | Absolute temperature | K |
| *x* | Distance to the electrode surface | cm |

**S2. ACTIVITIES FOR STUDENTS**

**Reversible electron transfers**

**1.** From Eqn. (12) of the main manuscript:

**a)** Derive the expression of the half-wave potential of reversible electrode reactions
(Eqn. (14)).

**b)** Derive the following expression for the current where the half-wave potential is taken as reference (instead of the formal potential):

$$\frac{I}{I_{\text{d}}}=\frac{1}{1+e^{\eta_{1/2,rev}}}$$

with:

$$\eta_{1/2,rev}=\frac{F}{RT}\left(E-E_{r}^{1/2}\right)$$

**c)** Derive the expression for the linear plot *E* *vs* $\ln\left(\frac{I_{\text{d}}-I}{I}\right)$ (Eqn. (13)).

**d)** Deduce the value of the cathodic ($E\to-\infty$) and anodic limiting current ($E\to+\infty$) and justify the results obtained.

*Hint*: Note that in the derivation of Eqn. (12) it has been assumed that only species O is present in solution.

**2.** From Eqn. (13), derive the following expressions for the potentials at which the current value is one quarter ($I=\frac{1}{4}I_{\text{d}}$) or three quarters ($I=\frac{3}{4}I_{\text{d}}$) of the limiting current:

$$E_{r}^{1/4}=E_{r}^{1/2}+\frac{RT}{F}\ln\left(3\right)$$

$$E_{r}^{3/4}=E_{r}^{1/2}-\frac{RT}{F}\ln\left(3\right)$$

from which the expression for the difference between them (Eqn. (15)) is obtained.

**Non-reversible electron transfers**

**3.** From the general solution for the current-potential response of electrode processes of any reversibility (Eqn. (19)):

**a)** Derive the expression corresponding to reversible processes (Eqn. (12)) as the limit when $k_{\text{red}}\to\infty$ and $k_{\text{ox}}\to\infty$.

**b)** Derive the expressions for the cathodic and anodic limiting currents and compare the results with those obtained for a reversible electrode reaction.

**4.** From Eqn. (21) for fully irreversible electrode processes:

**a)** Derive the expression of the half-wave potential of irreversible electro-reduction reactions (Eqn. (22)).

**b)** Taking into account that the exact expression for $E_{\text{irrev}}^{1/2}$ at macroelectrodes is given by

determine the absolute error of the approximate expression (22).

**c)** Derive the following expression for the current where the half-wave potential is taken as reference (instead of the formal potential):

$$\left(\frac{I}{I_{\text{d}}}\right)_{\text{irrev}}=\frac{1}{1+e^{\alpha\eta_{1/2,irrev}}}$$

with:

$$\eta_{1/2,irrev}=\frac{F}{RT}\left(E-E_{irrev}^{1/2}\right)$$

**d)** Derive the linearized plot *E* *vs* $\ln\left(\frac{I_{\text{d}}-I}{I}\right)$ (Eqn. (23)).

**5.** From Eqn. (23), for an irreversible process, derive the following expressions for the potentials at which the current value is one quarter ($I=\frac{1}{4}I_{\text{d}}$) or three quarters ($I=\frac{3}{4}I_{\text{d}}$) of the limiting current:

$$E_{irrev}^{1/4}=E_{irrev}^{1/2}+\frac{RT}{\alpha F}\ln\left(3\right)$$

$$E_{irrev}^{3/4}=E_{irrev}^{1/2}-\frac{RT}{\alpha F}\ln\left(3\right)$$

from which the expression for the difference between them (Eqn. (24)) is obtained.

**6.** From Eqn. (21), deduce the Koutecký-Levich equation for irreversible reactions at rotating disc electrodes (Eqn. (28)).

**Practical examples**

**1.** In the Excel file “SI - Practical example 1.xlsm”, the theoretical $I\text{ (A) }vs\text{ }E\text{ (V)}$ response is calculated for the irreversible reduction of Fe(III) on Pt, Fe(III) + e^−^ → Fe(II), for which *E*^0’^ = 0.771 V (vs SHE) and considering $D_{\text{Ox}}=D_{\text{Red}}=10^{-5}\text{ cm}^{2}\text{/s}$, $c_{\text{O}}^{*}=1\text{ mM}$ and *T* = 298 K. With the $I\text{ (A) }vs\text{ }E\text{ (V)}$ data corresponding the case of a planar electrode with A = 0.4 cm^2^ and $t=1\text{ s}$, perform the following kinetic analyses:

**a)** Verify the linear relationship between $E\text{(V)}$ and $\ln\left(\frac{I_{\text{d}}-I}{I}\right)$. On the basis of Eqn. (23), obtain the values of the transfer coefficient, $\alpha$, and of the standard heterogeneous rate constant, , via simple linear regression analysis.

**b)** Prepare the Tafel plot $E$ *vs* $\text{log} \text{(}I\text{)}$ at potentials corresponding to the foot of the wave (approximately 15% of the maximum current). On the basis of Eqn. (31), determine the values of $\alpha$ and via simple linear regression analysis of the data.

**c)** Compare your results with the values reported for the kinetic parameters of this system: *k*^0’^ ≈ 9 x 10^-6^ cm/s (25ºC) and α = 0.50 ^1^.

**2.** In the Excel file “SI - Practical example 2.xlsm”, the theoretical $I\text{ (A) }vs\text{ }E\text{ (V)}$ response is calculated for the irreversible reduction of Eu(III) on Hg, Eu(III) + e^−^ → Eu(II), for which *E*^0’^ = −0.160 V (vs SHE) and considering $D_{\text{Ox}}=D_{\text{Red}}=10^{-5}\text{ cm}^{2}\text{/s}$, $c_{\text{O}}^{*}=1\text{ mM}$ and *T* = 298 K. With the $I\text{ (A) }vs\text{ }E\text{ (V)}$ data for the following cases:

- Macroelectrode with *A* = 0.8 cm^2^ and $t=1\text{ s}$.
- Disc ultramicroelectrode with $r_{\text{d}}=\text{5 }\mu\text{m}$.
- Rotating disc electrode (RDE) with *A* = 0.8 cm^2^, $\omega=100\text{ rad/s}$ and $\nu=0.0091\text{ cm}^{2}\text{/s}$ (water).

perform the following kinetic analyses:

**a)** For all the above electrodes, verify the linear relationship between $E\text{(V)}$ and $\ln\left(\frac{I_{\text{d}}-I}{I}\right)$, and obtain the values of the half-wave potential. In each case, on the basis of Eqn. (23), obtain the values of the transfer coefficient, $\alpha$, and of the standard heterogeneous rate constant, , via simple linear regression analysis.

**b)** Justify the shift of the half-wave potential towards more negative values as the electrode size shrinks.

**c)** According to Eqn. (28), prepare a Koutecký-Levich plot, $1/I$ *vs* $1/\omega^{1/2}$, for the RDE (for example, in the range $10<\omega\text{(rad/s) < 500}$) at four different $E$-values: −0.250, −0.275, −0.300 and −0.325 V. For each $E$−value, obtain the corresponding $k_{\text{red}}$−value and then carry out the simple linear regression analysis of $\text{l} \text{n} \text{(}k_{\text{red}}\text{)}$ *vs* $E$ to determine the values of $\alpha$ and .

**d)** In all cases, compare your results with the values of the kinetic parameters of this system: *k*^0’^ = (1.4−1.7) x 10^-4^ cm/s (25ºC), α = 0.66 – 0.69 ^2^.

**S3. MARKING GUIDES**

**S3.1. Activities**

|  | **Criteria** | **Weight** |
| --- | --- | --- |
| **Organization and presentation** | - Solution is written in a clear and legible way. | 10% |
| **Mathematical manipulations** | - All necessary information is identified correctly. - Mathematical derivations are correct. - Progression from the first to the last steps is detailed. | 30% |
| **Variables and constants** | - Physical magnitudes and constants are identified and their symbols are correct. | 15% |
| **Results** | - The final expression is correct. | 25% |
| **Analysis of results** | - Most relevant findings and conclusions are identified. - There is evidence of critical thought process. - Results are compared with those in bibliography. | 20% |

**S3.2. Practical examples**

|  | **Criteria** | **Weight** |
| --- | --- | --- |
| **Organization and presentation** | - Report is written in a clear and legible way. | 10% |
| **Mathematical manipulations** | - All necessary information is identified correctly. - Mathematical derivations are correct. - Progression from the first to the last steps is detailed. | 20% |
| **Data analysis** | - Conditions of applicability of the equations are identified. - Graph axes are labelled and scaled appropriately. - Simple linear regression is performed and reported correctly. | 25% |
| **Variables, constants  and units** | - Physical magnitudes and constants are identified and their symbols are correct. - Adequate units are indicated in the text and graphs. | 15% |
| **Results** | - The final expression or value is correct. | 15% |
| **Analysis of results** | - Most relevant findings and conclusions are identified. - There is evidence of critical thought process. - Results are compared with those in bibliography. | 15% |

**S4. BIBLIOGRAPHY**

(1) Oldham, K. B.; Myland, J. C.; Bond, A. M. *Electrochemical Science and Technology: Fundamentals and Applications*; John Wiley & Sons: Chichester, 2012.

(2) Henstridge, M. C.; Laborda, E.; Wang, Y.; Suwatchara, D.; Rees, N.; Molina, A.; Martínez-Ortiz, F.; Compton, R. G. Giving Physical Insight into the Butler–Volmer Model of Electrode Kinetics: Application of Asymmetric Marcus–Hush Theory to the Study of the Electroreductions of 2-Methyl-2-Nitropropane, Cyclooctatetraene and Europium(III) on Mercury Microelectrodes. *J. Electroanal. Chem.* **2012**, *672*, 45–52. https://doi.org/10.1016/j.jelechem.2012.02.028.
